# Practical Analysis and Urinary Diagnosis

**Published:** 1895-01

**Authors:** 


PRACTICAL URANALYSIS AND URINARY DIAGNOSIS: A MANUAL FOR THE USE OF PHYSICIANS, SURGEONS AND STUDENTS. By Charles W. Purdy, M. D., Queen's University; Fellow of the Royal College of Physicians and Surgeons, Kingston; Professor of Urology and Urinary Diagnosis at the Chicago Post-Graduate Medical School: Author of "Bright's Disease and Allied Affections of the Kidneys;" also of "Diabetes: Its Causes, Symptoms and Treatment." With Numerous Illustrations, including Photo-engravings and Colored Plates In One Crown Octavo Volume, 360 Pages. In Extra Cloth, $2.50 net. Philadelphia: The F. A. Davis Co., Publishers, 1914 and 1916 Cherry Street.

It affords us great pleasure to commend the above work in the highest terms, for within its pages the physician and student will find accurate information on every point touching the urine and urinary diagnosis. The author has divided the work into two parts. The first part—"Analysis of Urine"—comprises General Considerations, Theories of Secretion and Excretion of Urine, Composition of Normal Urine, Abnormal Urine, Proteids, Carbohydrates, Urinary Sediments, Chemical Sediments, Anatomical Sediments, Gravel and Calculus. The second part—"Urinary Diagnosis"—is devoted to Diseases of the Urinary Organs and Urinary Disorders, the Urine in Other Diseases. The author includes under Urinary Diagnosis: "*First*, diagnostic data derivable from the urine, which relate directly to pathological conditions of the urinary organs themselves. *Second*, diagnostic data derivable from the urine, which relate to pathological conditions, either local or general, but the prominent feature of which is some marked and characteristic departure from the normal condition of the urine itself. *Third*, diagnostic data derivable from the urine, which relate to pathological conditions primarily independent of the urinary organs, in which the latter may or may not become involved."

The author reviews carefully the various methods of qualitative and quantitative determination of urinary constituents, both physiological and pathological, and calls especial attention to the inaccurate and misleading results obtained from many of the popular tests in vogue among the profession. For the detection of the presence of albumen, he recommends the *ferrocyanide* test as simple and free from sources of error, and altogether superior to the heat and nitric acid test as usually performed. For the detection of sugar, he advocates the use of *Haines' Solution* in place of Fehling's, as being quite as delicate and possessing the advantage that the solution keeps indefinitely.

For obtaining urinary sediments for microscopical examination, the author most strongly urges the great advantages of the centrifugal methods, being a saving of time and, what is of greater import, obtaining the sediment while the urine is yet freshly passed, thus avoiding all possibility of decomposition.

The illustrations are numerous and the plates excellent.

